# The necessity revealed by COVID-19 pandemic: Paradigm shift of Iran's healthcare system

**DOI:** 10.3389/fpubh.2023.1041123

**Published:** 2023-01-24

**Authors:** Mohammadtaghi Mohammadpour, Sajad Delavari, Zahra Kavosi, Mahmoudreza Peyravi, Reyhane Izadi, Peivand Bastani

**Affiliations:** ^1^Student Research Committee, School of Health Management and Information Sciences, Shiraz University of Medical Sciences, Shiraz, Iran; ^2^Health Human Resources Research Center, School of Health Management and Information Sciences, Shiraz University of Medical Sciences, Shiraz, Iran; ^3^Department of Health in Disasters and Emergencies, Health Human Resources Research Center, School of Management and Medical Informatics, Shiraz University of Medical Sciences, Shiraz, Iran; ^4^Department of Health Care Management, School of Management and Information Sciences, Shiraz University of Medical Sciences, Shiraz, Iran

**Keywords:** COVID-19, pandemic, paradigm shift, challenges, health system, healthcare system

## Abstract

**Background:**

COVID-19 pandemic has resulted in drastic changes around the world, revealing vulnerable aspects of healthcare systems. This study aimed to explore how Iranian healthcare system experienced the paradigm shift during the pandemic and determine the aspects that need improvement during the pandemic era.

**Method:**

This qualitative study was conducted in 2021. A framework analysis approach was used to analyze the content of the 19 semi-structured interviews with the healthcare system experts from Shiraz University of Medical Sciences (SUMS). The interviews‘ audio files changed into transcript after each session and data was saturated at the 19 interview. To increase the trustworthiness of the study, Guba and Lincoln's criteria including credibility, transferability, dependability, and confirmability were used. Goldsmith's five-step framework analysis was used applying MAX QDA version 10 software.

**Result:**

Eight main themes and 20 subthemes were explored. The main themes included “strengthening the electronic health infrastructure,” “research for evidence-based decision making,” “dedicated financing to the pandemic,” “prevention of disruption in the effective provision of services and medicines,” “enriching the authority of the Ministry of Health by focusing on interactions,” “recruiting, managing and empowering health human resources with attention to financial and non-financial incentives,” “reforming educational approaches in training students in medical universities,” as well as “lessons learned from neglected aspects.”

**Conclusion:**

To be ready to respond to a possible future pandemic and for a paradigm shift, bold steps must be taken to make fundamental changes in various aspects of the healthcare system including e-health development, evidence-based decision making, dedicated budgets for pandemics, reinforcement of interactions at the national and international level, as well as sufficient attention to healthcare workers from all financial, non-financial and educational aspects.

## Introduction

COVID-19 was announced as a pandemic on March 11, 2020 and resulted in drastic changes around the world with significant negative outcomes on all aspects of the population‘s life (1, 2). The pandemic‘s effects could not only be mentioned from the health perspective but also could be considered from its significant impacts on all other environmental, social, educational, and economic aspects of the communities (2, 3). Without a doubt, COVID-19 pandemic put the most pressure on healthcare systems, and this led to the revelation of less-than-optimal resilience of even high-performing healthcare systems (4). Such an unprecedented pressure almost put healthcare systems on the verge of collapse in many developing countries (5).

The possible imbalance between supply and demand factors intensifies the adversities and vulnerabilities of healthcare systems during any humanitarian crisis, and this has been acutely experienced during the COVID-19 pandemic, reflecting the vulnerability of healthcare systems in countries around the world. The pandemic apparently clarified that healthcare systems could be more vulnerable in the face of unequal demands (5). Some countries that were long thought to have the best or close to the best healthcare systems in the world seem to have realized after the outbreak of COVID-19 that they may have been wrong for a long time; because of this pandemic, the problems of their healthcare systems, including barriers to access, uncontrolled costs, unacceptable quality, and wide disparities, were clearly revealed (6). In fact, this pandemic imposed a serious pressure on the performance of the healthcare systems and as a result, many of these systems became profoundly unstable and lost their capacity of care due to a sudden and severe change or shock. At the same time, healthcare systems faced specific challenges, including disease burden and excessive mortality which led to delays in urgent non-COVID care (7).

Delays in routine healthcare during COVID-19 are a critical issue, not only because of the magnitude of delays that occurred during the pandemic, but also due to the sheer volume of delays in routine care. According to the evidence, colon and breast cancer screenings dropped by more than 80% and the healthcare delays negatively affected quality of life, morbidity, and mortality among the population (8). Another complication of this pandemic is the increase in the possibility of malnutrition due to quarantine and unemployment, which leads to the negation of the achievements of the national health and nutrition programs. But the more worrying aspect is the lack of proper safety nets (e.g., food safety) at least for the most vulnerable population (9).

Several factors led the healthcare systems toward this set of challenges and complications. On the one hand, fragility and unpreparedness of healthcare systems, lack of resources along with poor service delivery made the healthcare systems collapse during the pandemic (10). For example, in India, poor health infrastructure coupled with poor logistics led to severe oxygen shortages despite having excess oxygen capacity (5). Other evidence indicates the negative effects of the pandemic on healthcare services‘ delivery due to human resources‘ challenges. The main concerns in this field include the number, distribution, type, and performance of healthcare workers. It is quite clear that the optimal management of health human resources and the timely identification of related challenges in this field are the way for policymakers to better manage this pandemic (11).

Considering all the above, healthcare policymakers are seeking approaches to make the systems more resilient and flexible. Among them a four-stage life cycle of shocks is notifiable. According to this cycle, a healthcare system first tries to be prepared; identify onset and act rapidly. Then the impacts are managed to preserve healthcare system access and quality as well as dealing with legacy issues thereafter (7). Such approaches need an appropriate evidence-based decision-making mechanism based on strong research skills, the capacity to conduct accurate and rapid research, evidence evaluation, as well as structures for informed decision-making (12). Along with the set of solutions and approaches to increase the responsiveness of the healthcare systems, measuring, monitoring, and reporting performance can lead to a balanced responding mechanism to COVID-19 pandemic (13).

All in all, COVID-19 is not the first pandemic, and it will not be the last (14). Beyond defeating this pandemic, the big test that all countries will soon face is whether the lessons from this experience have shaped new shared goals after the crisis (6). Although this crisis revealed the need for a paradigm shift in public health policies (15), it has led to a paradigm shift to prepare for future crises (16).

Iranian healthcare system like other similar settings faced multiple difficulties during the pandemic. An ongoing challenge is coping with the pandemic condition along with the insufficient coordination between the internal and external sectors. This issue is mostly due to the lack of a proper crisis management plan. Among other challenges, one may mention transparency in information and building public trust. It seems the officials involved in this crisis have not adopted appropriate information policies and programs during this crisis. Sometimes the delay in providing statistics has led to increased society concerns and the creation of incorrect and false information. Also, in the past years, due to the treatment-oriented attitude of the Ministry of Health and the delay in the implementation of the family physician program, as well as insufficient attention to primary health care, the management of the COVID-19 pandemic has faced many problems (17, 18).

Considering the above, this research aimed to explore how Iranian healthcare system experienced the paradigm shift during the pandemic and determine the aspects that need improvement during the pandemic era. The present results could shed the light for Iranian health policymakers and those with similar settings to improve the performance, resilience, and responsiveness of the healthcare systems during the pandemics.

## Methods

### Study setting

This was a qualitative study conducted in 2021 applying a framework analysis approach. The study setting consisted of the experts affiliated with Shiraz University of Medical Sciences (SUMS). This is the largest and the most high-rank university in south of Iran and is considered as the referral system for many other universities all are affiliated with the Iranian Ministry of Health and Medical Education (MOHME). Considering the structure of Iranian healthcare system, MOHME is responsible for the population‘s health and policymaking at the national and local levels; all other medical centers and service providers, as well as research and educational centers, such as SUMS are supervised by MOHME.

### Study participants

The study participants, including those experts with administrative and practical experience in primary health care, health policy and healthcare system were selected. For achieving more comprehensive opinions, those academic members who had related scientific experience, related research and executive experiments were also included.

To increase the variety and heterogeneity of the participants, a purposeful sampling was applied and followed by a snowball sampling method. The inclusion criteria for the purposeful sampling were having at least 3 years' experience in the areas of primary health care, health policy making and healthcare system or health executive management and leadership as well as willingness to participate. Following the snowball sampling and in accordance with the introduction of the initial purposeful participants, other individuals who could contribute and help develop the concepts were recognized. The study's withdrawal condition was unwillingness to participate.

According to the described protocol for data collection, at the first step, two participants were selected purposefully (Head of Health Policy Center and Vice President of Health at SUMS). Then, 17 participants were included by snowball sampling who were all considered as the key health policy makers and leaders in the management of COVID-19 pandemic at SUMS. All of them had sufficient experience and information and were willing to participate. At this level, after 19 semi-structured individual interviews the data was saturated, and no new concept was generated.

Demographic characteristics including education level, specialization, management experience, age, and marital status were registered for the interviewees. The characteristics of the participants in the research are given in Table 1.

**Table 1:** Participants categories.

**Variables**	**Divisions**	**Frequency**
Position	Health policy makers	7
	Deputy health officials	4
	Faculty member	4
	Executive directors of the health department	4
Gender	Male	16
	Female	3
Working experience	< 10	5
	≥10	14

### Data collection

Semi-structured interviews were used to collect comprehensive insight from included experts by one of the researchers (MM) during December 2020 and January 2021.

To prepare the interview guide, a quick literature review was conducted as well as the opinion acquisition of selected experts. To ensure the meaningfulness and validity of the questions, two pilot interviews were conducted with the faculty members in the field of health policy who were not included as the main participants. The final interview guide included a warm-up question, and 12 main and sub-questions as well as probing questions.

The interview sessions were conducted with prior coordination with the participants at their favorite time and preferably in the workplace and face-to-face. At the beginning of the interview, general explanations about the study and its objectives, as well as the measures taken to keep their information confidential, were presented orally. Also, a written informed consent form was obtained from all the interviewees and the participants were assured that they could stop the interview process at any stage. With the permission of the interviewees, all the interview sessions were recorded and for more accuracy, their non-verbal gestures were noted. Each interview lasted at least 50 min and all interviews were conducted by one researcher (MM) and continued until reaching the saturation level. As soon as possible after the end of each interview, the audio recordings were transcribed verbatim. Since the interviews were in Farsi, the quotes were translated from the original by the study team.

### Data analysis

To analyze the data, Goldsmith's five-step framework analysis method was used (19).

° In the first step, for the purpose of familiarization, the audio recordings from the interview sessions were transcribed verbatim, and to ensure the initial and targeted understanding of the data, the texts were reread several times. During this step, by immersing in the data and taking notes on key ideas, the researcher began to understand the main themes in the data, and this step continued until the researcher felt a reasonable initial understanding had been reached.
° In the second step, the analysis moved from the concrete descriptions of the themes in the data toward the identification of more abstract concepts, and with the aim of providing a framework or structure for interpretation, the repeated ideas in the familiarization step became groups consisting of similar ideas. In fact, the themes and concepts were categorized and arranged in such a way that it helped the researcher to focus on the study and interpret the findings in an organized manner.
° After a reasonable framework was identified, this framework was systematically applied to all study data. In this process, which is called indexing, units and parts of data that were related to a specific theme were identified.
° In the fourth step, which is called charting, the data were summarized and tabulated based on the thematic framework, to provide the possibility of a totality and systematic examination of the data.
° Finally, in the fifth step, the data were finally combined. The researcher tried to tell a compelling story about how the data was structured and patterned, and used mapping to describe key concepts, their nature, or scope, and to show connections between key concepts.

All the data analysis process was implemented by two of the researchers with no conflict of interest. Reflexivity of qualitative data analysis was also assured as the research team members have sufficient experience in this field and have published several qualitative articles in English.

### Trustworthiness criteria of the qualitative study

To ensure the trustworthiness of the research data, the four criteria proposed by Lincoln and Guba, including credibility, transferability, dependability, and confirmability, were used.

To ensure the credibility of the study, the method of long-term participation and interaction between the researchers and the participants was used. To ensure the transferability, a thick description of the data was used. The research environment, the conditions of the participants and the interview method were clearly defined. Also, an in-depth description of the data, how to code and analyze movements and texts, was provided. And, to confirm the dependability of the research, the step-by-step repetition method was used to analyze and collect data. Finally, to ensure the confirmability of the research, cross-checking with other members of the research team was used, and expert check and peer check were applied for data accuracy.

## Result

The findings from the analysis of the interviews led to the identification of 8 main themes and 20 sub-themes (Table 2).

**Table 2:** Identified challenges that required changes in Iran's healthcare system.

**Theme**	**Subtheme**	**Code**
Strengthening the electronic health infrastructure	Health Information System‘s integration	Multiplicity of information systems
		Duplicate information
		Tiering data access
	Data accuracy	Correct analysis of data
		Enhancing the authentication process
		Data quality
	Comprehensiveness	Process for collecting data
		A lack of complete data reporting
		Health data integrity at all levels
	Security	Users with specified access
		User validation
		Improving the security of health information
Dedicated pandemic financing	Consideration of a dedicated pandemic control budget	Lack of specific budget line for crises
		The Ministry of Health does not adequately fund medical sciences universities in order to manage pandemics
		Hospital revenue reductions and related problems during the pandemic
	Reforming the health financing system	Payment system reform
		Transparency and efficiency in the allocation of special pandemic funds
		Health expenditures per capita are declining
Research for evidence-based decision making	Applied research in health and prevention	Expanding access to data for researchers
		Orienting researchers' attitude toward applied research
		Multidimensional analysis of evidence
	Preparing a national road map for research	Future research of infectious diseases
		Research needs assessment according to the health status of the society
		Presenting research projects in policy making committees
Prevention of disruption in the effective provision of services and medicine	Development and implementation of clinical guidelines	Evidence-based pharmacotherapy with a focus on strengthening guidelines and training
		Update according to the change of disease variants
		Formulation of guidelines using health technology assessment
	Improving service delivery	Preventing service delivery disruptions for other diseases by focusing on public education
		Private sector participation
		Priority of prevention instead of treatment
		Modifications to the basic package of health services
	National diseases surveillance system	Strengthening the laboratory network
		Infectious disease syndromic improvement
		Infectious disease control center management needs to be strengthened and improved
		A clear report on the side effects of vaccines
Enriching the authority of the Ministry of Health by focusing on interactions	Restructuring Health Ministry decision-making and policy	Resolving multiple voices in decision-making at the national level
		Decentralization of decisions at the regional level
		Decision-making conflict of interest
	Strengthening national and international interactions and advocacy	Use of non-governmental organizations
		Strengthening the health ambassadors' program
	Coordination between the internal and external sectors	Horizontal integration in service delivery
		Vertical integration in service delivery
		Improve referral levels
		Strengthening external-sectoral coordination with a focus on increasing the power of MOHME
	Social marketing in COVID-19	Attracting public trust
		Public participation
		Society's obedience to the laws
Recruiting, managing and empowering health human resources with attention to financial and non-financial incentives	Continuous education of health sciences	Expansion of specialized training related to the pandemic
		Creation of crisis education core
		Continuation of “health in disasters” training all the time
	Strengthening the financial benefits of the health workforce	Payment problems
		Job insecurity
		Allocation of financial rewards for frontline health worker
		Process facilitation for staff
		Communicating with employees in a standardized manner
Reforming educational approaches in training students	Alignment of education and the health system	Changing and updating educational protocols
		Changing the curriculum of medical education according to family medicine
		Health faculties should incorporate a health department
	Changing the practice of health students due to the effects of the pandemic	Revealing the necessity of field work for students
		Pandemic as a golden opportunity
		Development of virtual education
Lessons learned from neglected aspects	Unused capacities, especially the primary health care system	Increasing active centers providing primary health care services
		Task shifting
		Paying attention to the role of the health network in promoting the health literacy of the community
	Planning and modifying infrastructures to deal with possible future pandemics	Completing the organizational positions of the healthcare network system
		Using epidemiologists in the health network
	Strengthening the Center for Infectious Disease Control and Prevention	CDC strengthening its position in the accreditation of health care facilities
		Modifying organizational charts related to the management of infectious diseases
		Inefficient policies

### Strengthening the electronic health infrastructure

Strengthening the electronic health infrastructure includes four sub-themes as follows: integrating the health information system, data accuracy, comprehensiveness data, data security The interviewees believed that the e-health infrastructure should be strengthened to generate evidence and provide services. One of the participants as a primary health care manager with more than 10 years of experience stated:

“*...for example, we have a SIB system)* Integrated health system *(, on the other hand, we have several portals where information has to be recorded in several places, repetitive and sometimes unnecessary information, which makes it impossible to report correctly and on time, and the accuracy of the information was also a problem*...” [P5/ deputy health officials]

Another participant expert in health policies said:

“*At all, the SIB)* Integrated health system *(cannot be considered as an electronic health file, the SIB system, which the information of the MOHME is leaked with the ID number, the most obvious characteristic of a system is that it has information security”*.

The interviewees believed that the existence of a database facilitates the evidence-based decision-making process. One interviewee stated that:

“*The most important thing in a pandemic is a decision based on evidence. Evidence means data and information, and according to the famous pyramid that we know, at the bottom of that data, next is information, knowledge, and then decision”*

One of the concerns raised in this study was improving health information systems so that they provide optimal information dashboards for policy makers, researchers, and managers. As an example, one of the participants with experience in health policy making stated:

“*... important information should be available to managers for decision-making, and the prerequisite for that is the creation of a broad and integrated information system*” [P11/ health policy makers].

As a result of this research, one of the issues raised was the strengthening of digital health infrastructure. Despite the seriousness of this issue around the world, it appears that digital health structures in Iran are still incomplete and underdeveloped. one of the participants with experience in health policy making stated:

“*Another discussion is that the future of health systems is going toward digitalization. This is a very important discussion. Meetings are held in European countries, and they are looking to strengthen telehealth and compensate part of the weakness of their system in this way, and they are preparing... Now you come and design a system for this purpose, did you work in a forward-looking way? No. Because the systems are tools and the infrastructure of manpower, training and equipment must be provided first...”*

The participants believed that information systems should be sensitive to statistics and give automatic warnings about risks. In this regard, one of them said:

“*Creating a smart information network for timely diagnosis of epidemics and diseases in the country for the post-pandemic era can be very helpful*” [P14/ health policy maker].

### Research for evidence-based decision making

Research for evidence-based decision making includes two sub-themes as follows: Applied research in health and prevention, preparing a national road map for research. A participant stated that:

“*This restricted access to data must be resolved. When the data is collected, it must be thought of for its dissemination, that is, it must be leveled, and each part can have specific access to the data. MOHME should have one level of access while the researchers need another level of access and similarly for the public, otherwise we may make wrong decisions and people will see the harm”* [P5/ university professor*]*.

Many of the interviewees believed that research should be purposeful and lead to decisions. A participant stated that:

“*During COVID-19, the research departments had the least cooperation and preparation, and in this field, now that we have passed the peak of COVID-19, it should be thought about making research more practical*” [P16/ Faculty member].

In the field of health research, it is a challenge that less attention is paid to the practical aspects of research. Despite conducting applied research, the results may not be incorporated into health policy formulation. For making health policy decisions, there may not be a scientific roadmap. In post-COVID-19 era, it could be considered, According to one of the participants with academic and managerial experience in the COVID-19 crisis:

“*... actions must be taken, one of them is decision-making based on scientific evidence, for this we must have a research mapping, that is, in research, we must first extract specific questions that are necessary for policymaking, then we will turn these into a proposal or an RFP order, then these will turn into a series of scientific proposals, and these proposals must come to the scientific committees and the scientific committee will turn them into policies*.

The participants stated that it is necessary to analyze the statistics with a multi-faceted perspective and decide about the risks may impose on society. One of them said:

“*The next point is the lack of proper data analysis. If we do not analyze the data correctly, we will make an error in the conclusion. Statistics need technical analysis, and it is not the work of one person, and it should be looked at from different perspectives… we need to have the right indicators for risk assessment, that is, to make a wise decision according to the level of risk in the society, if we can control the risk by closing one class, there is no need to close all schools*” [P11/ health policy maker].

### Dedicated financing to the pandemic

Dedicated financing includes two sub-themes as follows: consideration of a dedicated pandemic control budget and Reforming the health financing system. Most of the participants stated that during COVID-19 pandemic, the MOHME did not have enough financial resources and these minimal resources were not properly allocated.

“*...we did not have money, the money we earned was given to us in installments, unfortunately, it came too late and lacked transparency, lack of timely allocation, financial corruption occurred and even the allocation was not efficient”*

Participants agreed that MOHME‘s budget should include a specific line item for health crises. A participant with work and research experience in primary health care stated:

“*There is no budget under the title of crisis management in MOHME, whatever it is, this budget is current, and you should allocate the money that comes for normal times to the crisis*” [P10/ executive director].

There are two main payment systems in Iran's health system: fee-for-service and fixed salary payments. This is one of the most significant financing problems in Iran's health system. There are numerous evidences of the inefficiency of this system, yet there is no willingness to correct it. This can be attributed to the treatment-oriented approach and the absence of evidence-based policymaking in the Ministry of Health's executive decisions. Likewise, health expenditures per capita have decreased in recent years. In this regard, one of the participants with experience in policymaking said:

“*… the first factor was the sanctions and the economic undersecretaries. In the World Bank category, I dropped from the higher middle-income category to the LMC category as our GDP dropped. On the other hand, the payment system is inefficient at the specialized levels and does not provide the necessary efficiency. The group related to diagnosis has been proposed for years, but it is still being implemented incompletely. It seems that one of the most significant decisions of health policy makers in the post-Corona era should be the reform of the payment system”*.

### Prevention of disruption in the effective provision of services and medicine

This theme includes three subthemes as follows: Development and implementation of clinical guidelines, Improving service delivery and National diseases surveillance system.

The interviewees believed that the unavailability and irrational prescription of important medicines were the main challenges of the COVID-19 crisis. One of the interviewees said:

“*…sometimes medicines like Favipiravir were proven ineffective, but they were still prescribed or used. It seems that we did not have proper guidelines in this field, or the treatment staff was not given full and timely training; Or, for example, remdesivir is effective only in the viral phase of the disease, but we saw that it was prescribed for everyone, which would have caused a waste of resources, and its complications and side effects would have been problematic” [P9/ health policy maker]*.

Another challenge during pandemic management was the inefficiency of some medicines for COVID-19 that would be covered by insurance. Nevertheless, the financial problems in the pandemic were exacerbated by spending on imported medicines that were not in compliance with international guidelines. A participant with experience in health policy said:

“*Among the problems were the high cost of medicine and the lack of full support from insurance. The real need was more than the stock and caused the creation of a black market, so that sometimes a person had to spend more than several times the actual cost for medicines. The problems of economic sanctions should also be added to this issue, because of the sanctions, our access to some raw materials and medicines was limited”*

Another major challenge was providing services simultaneously for those suffering from COVID-19 and other diseases. One of the participants said:

“*We did not have dedicated human resources for this issue, so I did task shifting, for example, Behvarz* (multi-professional rural health worker of Iran's health system*) who are the main foundations of care, we had to reduce some of their tasks and duties, and shifted them specifically to COVID-19 for rapid identification, isolation and formation of rapid response teams... as a result, many of our routine services were disrupted, for example, we saw a drop in diabetes and blood pressure care”* [P17/ deputy health official].

One of the other participants claimed:

“*According to the studies conducted at Shiraz University of Medical Sciences, it was found that the services of pregnant mothers, children and diabetes have been disrupted and referrals have decreased compared to before the pandemic, which is partly due to people's fear of going to medical centers and...the solution that can be found in this field is one to get help from the private sector to provide services and second is to educate people about the need to refer and receive essential services such as routine vaccination or care of pregnant mothers”* [P15/ Faculty member].

Another sub-theme mentioned by the interviewees was focusing too much on treatment and neglecting prevention. A participant mentioned:

“*If you check the countries that are successful in this field, you will see that they can control the pandemic and reduce the burden of hospitalization by relying on extensive testing and active disease detection. This required allocating resources to the health department, which unfortunately was not done*” [P12/ Faculty member].

The participants believed that there should be a monitoring system of the community's health status in the field of communicable and non-communicable diseases, so that changes can be monitored at regional and national levels.

“*It seems that in the structure of the health network, there is a need to create a monitoring department, responsible for monitoring infectious, non-infectious and occupational diseases*” [P10/executive director].

Another participant added:

“*If the monitoring center is established in the infectious diseases management unit, it can be a successful policy for the post-COVID-19 era and facing the next pandemics” [P19/ deputy health official]*.

The lack of well-equipped laboratories in the provinces was one of the problems faced by the Iranian health system during the pandemic. In this way, laboratory tests for COVID were sometimes delayed and the golden opportunity to control and prevent the disease was lost. Participants with executive management experience in the field of health and prevention expressed an interest in this topic.

“*The main laboratory was all in the center, and to diagnose new cases, samples had to be sent to the center, which made the care identification process very difficult. It seems that one of the most important actions of MOHME after the pandemic should be to equip and improve the country's laboratory network”*

### Enriching the authority of the Ministry of Health by focusing on interactions

Enriching the authority of MOHME by focusing on interactions includes four subthemes as follows: Restructuring Health Ministry decision-making and policy, Strengthening national and international interactions and advocacy, Coordination between the internal and external sectors, Social marketing in COVID-19.

The participants believed that MOHME did not have enough political power to make effective decisions. One of them said:

“*The most important challenge of governance is the lack of unanimity and lack of focus in decision-making. This means MOHME should have full responsibility and all departments related to the pandemic should be subject to the Minister‘s decision, which was a serious challenge during the COVID pandemic. For example, there was not much consensus on vaccination or closing schools and universities, and the opinions of MOHME were not considered significantly” [P18/policy maker]*.

While there is a need for unanimity at the national level, some of the interviewees pointed out the necessity of decentralization for regional decisions, one of them said:

“*All decisions regarding the health of regions should not be made by MOHME and can be delegated to the regions, this top-down decision-making structure is very problematic... if MOHME wants to act centrally, it will not succeed in controlling epidemics, because Iran has different cultures, behaviors, and climates. Someone in Tehran should write a version that will benefit both Sistan Baluchistan and Fars, it is not answerable and useful, regional management and decentralization should be on the agenda” [P14/ health policy makers]*.

One of the major weaknesses that the interviewees agreed upon was the lack of internal and external coordination in the Ministry of Health, one of the participants stated:

“*Regarding coordination, I have a negative opinion, that is, coordination within the health sector, that is, between different levels, i.e., coordination between primary care and our treatment levels should exist... Of course, it cannot be said that there is a lack of coordination, but it can be said that the communication was not effective, this communication should be discussed in the form of structure and process, it was like this before the pandemic, neither our structure is basically a continuous structure, nor are the processes defined continuously*” [P14/health policy maker].

Participants reported that one of the structural weaknesses in the management of the pandemic was the lack of appropriate external coordination. Therefore, it was believed that MOHME was not able to ensure that policies were being implemented effectively.

Due to this situation, preventive policies are not implemented, such as gathering or traveling bans, or even complying with protocols such as wearing masks.

“*The most important lesson learned is that the responsibility of MOHME should be strengthened. In the beginning, the National Corona Headquarters was formed by the Minister of Health, all organizations had to come, several meetings were held. But they did not pay much attention to the words of the Minister, and at the end, the President himself became responsible for the National Corona Headquarters, because the influence of MOHME is weak” [P19/ deputy health official]*.

Someone else added:

“*Even we do not have intersectional collaboration, which means that there is no necessary coordination between the health system and other systems outside. This coordination was not there before; it only got worse during the pandemic*” [P17/ deputy health official].

The lack of advocacy at the national level and the weakness in international relations was a critical challenge in the recent pandemic. One of participants claimed:

“*SUMS was praised many times for its management, the reason was that the management of the university had very good interactions and was able to attract the necessary advocacy*” [P5/ university professor].

Another participant stated that:

“*Our international relations have problems in the field of epidemics, now we find that everything we have measles is from Afghanistan… for example, if you live in America now, they will give you a list of diseases of a certain country, get these vaccines, take these medicines with you, it means strong communication that gives them an exact list of health hazards*” [P7/ executive directors].

The need to gain people's trust and participation was another sub-theme mentioned, one of the interviewees said:

“*Transparency is very important, we did not explain to the people why, for example, the schools should be opened, we should show the evidence to the people, if this happens, I think the people will accept and support*” [P1/ university professor].

Similarly, another participant maintained:

“*You should inform the people about the danger that threatens them, this will enable people's participation”* [P11/ health policy maker].

Governance transparency is one of the most effective management tools during pandemics and similar crises. If used correctly, it can contribute to the successful implementation of health policies. In this context, one of the participants stated:

“*In a pandemic, there is an important issue under the title of risk communication and community participation, which means that you should inform the people correctly about the danger that threatens them. You can provide risk communication and the possibility of public participation. This requires building trust and transparency. There was no single spokesperson in MOHME, and different voices from the Ministry of Interior and the MOHME were heard in this field. People were confused about this. It used to be that about 30 people in some way announced themselves to MOHME and the health system and made comments, and this led to confusion... As a result, people lost trust, whereas they had strong trust at the beginning of the disease outbreak. We published a study in March 2018 that showed people have the highest level of trust in radio and television regarding Corona virus transmission but were unable to maintain this trust*” [P8/ Faculty member].

### Recruiting, managing, and empowering health human resources with attention to financial and non-financial incentives

This theme includes two subthemes as follows: Continuous education of health sciences, Strengthening the financial benefits of the health workforce. The optimal allocation of human resources and the use of specialists in their positions is one of the challenges of Iran's health system in the field of human resources. Meanwhile, temporary contract workers and their lack of job security are also among the concerns of human resources in the health sector. In the post-Corona world, one of the most important actions of the Ministry of Health should be to complete the organizational charts by using permanent personnel and paying attention to occupational health issues. One participant pronounced:

“*Not only in the case of COVID-19, the use of human resources in the entire health system is not optimal. For example, look at the central building of the university, more than 350 nurses and doctors are working in the administrative area! That is, instead of being in the treatment and service department, they are doing simple administrative work*” [P2/ health policy maker].

Furthermore, a participant stated:

“*One big challenge is the supply of forces, and the other is the quality of the forces. And let's divide the human forces into two categories, the forces that can work professionally in a pandemic, such as a nurse who has worked in the respiratory department and has expertise in this field, or a lung or infectious disease specialist, and the second category is general forces”* [P13/ health policy maker].

Another participant maintained:

“*The important point in this pandemic was the lack of training for these forces. One of the necessary tasks and plans is training of these forces*” [P6/ deputy health official].

In addition, another participant stated that:

“*We should invest especially on pandemics and crises management…. this issue should be part of continuous education in the health department and should not be neglected*” [P14/ health policy maker].

Another participant continued:

“*The second issue is motivation for human resources, which should be given special attention. For example, during the COVID pandemic, many of the personnel were not appreciated as they should be, and immediately after the COVID subsided, sometimes even their contracts were not renewed, or they were even given very little bonuses”* [P8/ Faculty member].

### Reforming educational approaches in training medical students

This theme includes two sub-themes as follows: alignment of education and the health system and changing the practice of health students due to the effects of the pandemic. One of the issues raised in this study was the strengthening of public health and related academic disciplines. The participants believed that this strengthening should be done in the context of changing the curricula of academic disciplines of public health and merging parallel educational centers in order to increase the influence of health faculties in the field of prevention and public health.

“*We must start the change first from the university and the health fields. It has been more than a decade now that the curriculum of the public health field, which is the executive arm of the health network, has not been updated”*.

In this regard, one of the participants with academic and research experience in primary health care stated the following:

“*Unless the students work at the bedside, they can't become a doctor. They can't be a driver until they sit behind the wheel, in health sciences, no one can claim to know the work without being in the field. I think that systematic planning should be done by health faculties for the participation of students in the field to really learn. Being in the field is a golden opportunity and an unforgettable experience”*

The alignment of education and the health system, as well as the integration of parallel education centers into the network system, was identified as a sub-theme. One participant said:

“*In the health network system, we have a training center for behvarz* (multi-professional rural health worker of Iran's health system)*, but these centers are not connected to health schools. By integrating these educational centers into university, one of the basic changes can be achieved in improving the scientific and academic level of this group. In contrast, Health Vice-Chancellors have practically no relationship with health colleges, even if the forces trained in the colleges are ultimately recruited into these vice-chancellors and subgroups. To improve the presence of students in the field and to strengthen the network system of Health Vice-Chancellors in colleges, it is important that health is integrated” [p6/ deputy health official]*.

One of the sub-themes identified was changing the education and training methods of medical students based on PHC:

“*We train medical students with a hospital approach, but we have no basic training in prevention and family medicine at universities, so we bring these physicians to health centers and expect them to work according to PHC goals. Changing the curriculum of medical education or training general doctors for family medicine from the beginning would be a good idea”. [p15/ Faculty member]*

### Lessons learned from neglected aspects

Learning lessons from neglected aspects includes three sub-themes as follows: Unused capacities, especially the primary health care system, planning and modifying infrastructures to deal with possible future pandemics and strengthening the Center for Infectious Disease Control and Prevention. As one of the main management units during the pandemic, the Center for the Control and Prevention of Infectious Diseases of the MOHME did not play a prominent role, it seems that the unit has been weakened by some wrong policies over the past few years, and serious reform is needed.

“*To deal with COVID-19, we needed a series of basic infrastructures that we did not have, such as a preparedness plan for pandemics, crises, and general epidemics. To do this, the Centers for Disease Control and Prevention must be agile, dynamic, and strong. It is not too late because our country is in an area that could experience other epidemics in the future*” [P11/ health policy maker].

Regarding strengthening the Center for Control and Prevention of Infectious Diseases, one participant said:

*One of the basic measures in the post-COVID era is to strengthen the management of infectious diseases. Our infectious disease control and prevention center has been weakened and neglected for various reasons before the outbreak of COVID-19. In my opinion, policy makers should prioritize this issue* [P9 health policy maker].

Despite regional conditions, policies at the Center for the Control and Prevention of Infectious Diseases weakened its subdivisions. One of the wrong policies before the pandemic was changing the organizational charts of the first-level service centers. These decisions did not seem to be based on scientific evidence:

“*It was a mistake to change positions, such as removing disease experts, in the health and treatment network. Our health policy makers believe that noncommunicable diseases are the main challenge we face, and vaccines and epidemiological trends have ensured that communicable diseases aren't a problem, but the COVID pandemic proved this wrong”* [p10/ executive director].

The participants believed that, although the overall capacities to respond to the COVID-19 crisis were low, these minimal capacities were not properly used. One of the interviewees claimed:

“*Sometimes we even saw that up to 20% can be added to the service delivery capacity of a treatment unit, so managing resources in a crisis is of particular importance”* [P15/ Faculty member].

Similarly, another participant said:

“*The capacities of the government and hospital sectors were full, and patients were dying because of this, and they could not coordinate the capacities of the private sector to come and provide services*” [P11/ health policy maker].

Another major challenge was inefficient decisions and policies, one of the interviewees said:

“*The control policies announced by MOHME were practically not a deterrent, for example, the fines considered for inter-provincial traffic were small amounts that did not have the ability to deter and control and were practically ineffective*” [P16/ Faculty member].

In general, the interviewees believed that Iran is a high-risk country and should be prepared to face the next possible crises both in terms of infrastructure and planning. A participant said:

“*The structure that is ready to face the crisis was not designed even on paper, for example, the laboratories were all in Tehran, that is, only one laboratory of the Pasteur Institute had been seen for these situations and there was none anywhere else in the country”* [P4/executive directors].

Besides, a participant asserted:

“*We wanted a series of basic infrastructures that we did not have, such as the readiness of our health system to deal with pandemics and crises. This issue requires an agile, dynamic, and strong CDC, up-to-date and with dynamic thinking. It's still not too late because our country is in a region that has the chance of encountering other epidemics. Our country is the meeting point of three epidemiological zones, which means we are at risk, the variety of diseases is high, we are not in an interesting situation from a geopolitical point of view, illegal traffic is rampant in many borders” [P2/ health policy maker]*.

## Discussion

The results showed that the areas that need to be improved in Iran's healthcare system because of the COVID-19 pandemic can be expressed in eight main themes. Each of these themes are discussed below to show the paradigm shift‘s requirements in Iranian healthcare system.

### Strengthening the electronic health infrastructure

The present results explore the necessity of the creation of a comprehensive and integrated health information system with accuracy and data security. Other evidence also emphasizes that the integrated electronic health record environment can provide a basis for consistent and verified access to basic information through the Internet to support decision making (20). Another study emphasizes that the privacy of patients and the security of their information is the most necessary obstacle for the adoption of e-health, and it points to the necessity of security techniques at three administrative, physical, and technical levels (21). Despite the importance of electronic health record data, less attention has been paid to the quality of the data. Keshta et al. in a study with the aim of evaluating the quality of the records of COVID-19 patients in the health information system, explored that ICD-10 codes were incorrectly assigned to the records of 238 patients (72.56%). More attention to data quality assessment as a prerequisite for patient safety and data readiness for research and predictive analysis, along with training healthcare providers about the importance of accurate documentation were among their recommendations (22).

Other results of this study emphasize providing a database with a certain level of access for decision makers at different levels with the aim of facilitating the evidence-based decision-making process. As other studies imply Ministries of Health in low- and middle-income countries often do not have access to high-quality and timely data. Lack of an organizational culture for data-driven decision-making is reported as well (23, 24). Data-based decision-making at different levels and in different areas of healthcare, including planning, procurement, and operations, providing health care between individuals, can effectively determine priority decisions (25).

Other related results in this area refer to the necessity of strengthening telehealth infrastructure. Telehealth is an efficient solution for healthcare delivery and has the potential to address many health systems challenges. But before the shaking of the health systems by COVID-19, little attention was paid to the implementation of telehealth. The evidence indicates that the implementation of this strategy requires the strengthening of infrastructures at four political, technological, organizational, and individual levels (26).

And the last result here is related to the use of intelligent systems for decision making. The present results clarify the need to use such systems in the field of clinical and political decisions. Other studies have also shown that intelligent systems can help decision makers improve the effectiveness of their decisions by integrating data mining techniques and model-based systems (27). In addition, other researchers emphasize that the use of Intelligent Decision Support Systems (IDSS) can have powerful help in solving difficult problems. These tools help overcome cognitive limitations and human biases and provide logical support to decision makers (28).

### Research for evidence-based decision making

One of the most fundamental challenges in the evidence-based decision-making process was the limitations of data access for researchers. In this regard, other studies have also pointed to the challenges of sharing health data with researchers during the COVID-19 era, which can lead to potentially harmful effects for citizens. The most important obstacles identified were legal conflicts between fundamental rights and data protection laws (29). Similarly, another researcher stated that one of the limitations of evidence-based decision-making in Iran is the lack of access to evidence (30).

Other results of this area were related to promoting the attitude of conducting applied research among researchers. Relevant evidence shows that the existence of research topics that generate sufficient interest in both the research and the policymaking communities and constructive collaboration among them may lead to increase the probability of conducting applied research and integrating research evidence into policymaking (31).

Preparing a national road map for research is among other results here. Other researchers emphasize that the 21^st^ century is the era of expansion of infrastructure and research roadmap. By using the research roadmap, it is possible to understand knowledge gaps, determine the direction and perspective of national research, reflect emerging research opportunities and challenges, provide important national facilities and services to support research and innovations, and help government decisions (32, 33).

Detailed data analysis with a multifaceted approach to determine the level of risk is mentioned among other results of the study. Similarly, another study showed that due to the complex nature of COVID-19 and its different effects on different groups, the response to the crisis should be accompanied by systemic thinking and a multifaceted approach with the participation of different organizations and people (34). In this regard, many other studies implied that due to the multifaceted nature of COVID-19, there should be a multifaceted treatment approach in the risk assessment and treatment of this disease (35). And similarly, a multidimensional approach should be taken in evaluating the fears related to this disease in society (36). Decisions must be accompanied by a holistic approach, an approach that integrates biomedical, psychoeducational, sociocultural, and justice perspectives. Applying this balanced approach to decision-making will help increase consistency and ensure that all viewpoints and concerns are considered (37).

### Dedicated financing to the pandemic

Among other challenges identified in this research which require particular attention, we can refer to the lack of special budget for pandemics in Iranian‘s healthcare system and lack of transparency in the resources spent in this area. Evidence shows that in response to the COVID-19 pandemic, many countries have reprogrammed their existing budgets while others have activated emergency reserves, considered supplementary budgets, or created extra-budgetary dedicated funds. There are various motivations behind the creation of these funds, one of which is to separate the costs of COVID-19 from other expenditures, thereby increasing financial transparency and accountability, and creating a well-defined audit trail. It has also been suggested to use certain performance indicators to evaluate the economic impact of EBFs (extra-budgetary funds) (38, 39).

### Prevention of disruption in the effective provision of services and medicine

Disruption in the provision of health services not only to COVID-19 patients, but also to other non-COVID-19 patients was another challenge. Restrictions on medical access and the creation of a drug black market due to economic sanctions have been considered as one of the fundamental challenges in the Iranian healthcare system in recent years, which worsened during the COVID-19 pandemic. Other evidence also shows that the sanctions have faced about 6 million Iranian patients with limited access to treatment and vital medicines. As a result, an unregulated black market has been created to compensate for drug shortages, offering drugs whose origin and authenticity are often unknown, expired, and sold at a much higher price than the actual price (40).

The next result refers to prescription without scientific support, which shows weakness in training and developing guidelines. Even before the beginning of this crisis, studies have shown that a very large percentage of therapists in Iran do not have access to databases for evidence-based medicine (41, 42). Also, the previous evidence indicates the weakness of Iran's healthcare system in the development, localization, distribution, and implementation of clinical guidelines (43). This age-old challenge has worsened in the era of COVID-19. Another study has also confirmed that evidence-based medicine has been shaken during this pandemic and doctors' trust has been eroded due to disagreements in scientific evidence and the publication of misleading scientific articles about COVID-19 (44).

Another identified weakness that needs to be strengthened was the disruption of services to other non-COVID patients. The results of this study indicate that by using existing potentials and changing procedures in the government sector, as well as seeking help from the private sector and increasing public awareness, this problem can be solved to an acceptable extent. Other studies have also shown that other non-COVID cares, especially routine ones (not emergency), were the most vulnerable, and had the most cancellations or postponements (45, 46). The shift of resources toward COVID patients and the lack of hospital beds, the diversion of staff from normal services to COVID services, along with the high public fear and anxiety of crowded places, all led to limited access to other non- COVID health services (47, 48). In response to this challenge, some centers divided their personnel into employees in COVID-19 zone and employees in COVID-19 free zone. Some centers, relying on the same existing resources, have used telemedicine as a tool to provide services and protect patients against COVID-19 (47, 49). The solution of some countries was to use the capacity of the private sector to access COVID-19 services, especially laboratory services (50). In general, governments' strategies to engage with the private sector developed rapidly, however, monitoring was often weak, indicating the weakness of governments in ensuring cost-effective and high-quality private services (51). To reduce the raised fear and concerns, the use of targeted training to deal with fear, and the expansion of mental health support have been suggested (52). In this regard, some studies have pointed out the influence of the media on increasing people's awareness and its role in the healthy behaviors needed in the era of COVID-19 (53).

The study participants emphasized the need for a prevention-oriented approach, so that this issue is considered in health policy and practice. Practical aspects of this approach include allocating financial, human, and physical resources to health networks and strengthening their infrastructure. This problem has already existed, and it became very troublesome during COVID-19. The healthcare system in Iran has a treatment-oriented approach, and prevention ranks second. Therefore, the priority of the healthcare system is to provide and increase the number of hospital beds, and community-oriented care and disease prevention have no place (54). While efforts focused on reorganizing and strengthening hospitals, primary and community care was largely neglected. Evidence shows that even the best hospital system could not cope with the demand caused by COVID-19 and social and home primary preventive care would have led to the reduction of this epidemic. As an example, Greece pursued the strategy of focusing on hospital preparedness but failed to strengthen primary care. This condition along with the country‘s strategic mistakes in epidemiological surveillance led to a case with the highest death rates from COVID-19 in Europe during the second wave (55).

Another identified weakness that needs to be strengthened is the lack of a specific structure in Iran's healthcare system to monitor the status of communicable and non-communicable diseases. As evidence in many countries suggests disruption of services to non-COVID patients (56, 57), particularly patients with non-communicable diseases (58), the present interviewees believed that this neglect is problematic and other diseases should also be taken into consideration in the national response to COVID-19. This requires the existence of a monitoring structure to determine the status of these diseases. The World Health Organization (WHO) considers it necessary to create this system for a comprehensive response to the pandemic, because it provides the possibility of monitoring vulnerable or at-risk populations and tracking epidemiological changes, which leads to effective response (59). On the other hand, delays in chronic care treatments, high numbers of deferred surgeries, and increases in mental health problems suggest a less visible epidemic that is quietly spreading and destroying people's lives, while COVID-19 gets all the attention (60, 61). Maybe a comprehensive surveillance system for other diseases can partially reveal and control the silent and possible pandemic ahead.

There was a mention of the weak laboratory network in this study, which requires an extensive infrastructure. Other studies have also pointed to the weakness of laboratory services during the COVID era due to lack of equipment because of sanctions, weak infrastructure, and laboratory scientific knowledge in Iran (62, 63).

### Enriching the authority of the Ministry of Health by focusing on interactions

One of the mentioned issues is solving the multi-voice problem in macro-decisions. Regional differences and the necessity of decentralization in regional decisions should not be forgotten. Although to make an effective decision, the preferences of all stakeholders should be considered and multi-voice decision making should be used (64), it is necessary to be aware of the complex role of powers in political processes and consider mechanisms to manage the influence of these powers, in a way that political interactive dialogues are directed toward strengthening priority programs (65). Another study also pointed to the complex network of key decision makers in this pandemic and the influence of international norms and political competition. In this regard, to control conflicts, increase legitimacy and protect against mismanagement, and increase the effectiveness of decisions, some countries have organized the National Committee for COVID-19 consisting of related ministries and headed by the Prime Minister, and they formed similar committees at the regional level (66).

The next sub-theme refers to advocacy and increasing political interactions at the national and international levels which can facilitate the previous sub-themes. Iran's experience in recent years shows many challenges in the healthcare system due to weakness in international political relations and sanctions (67). Since the beginning of the pandemic, the necessity of determining the key principles of international relations and attracting political support at different levels has been pointed out, and “lack of leadership and solidarity at the global and national levels” has been introduced as the biggest threat of this pandemic. In this regard, WHO, like an international magnet, strengthened relations between countries and controlled governments by developing international health regulations, and connected global and national reactions and decisions with different interactive patterns (68).

In the next sub-theme, the internal coordination of the healthcare system and the strengthening of the referral system are mentioned. To ensure the effective implementation of measures, WHO recommended the necessity of careful monitoring of service delivery patterns–especially for essential health services- and coordination with relevant authorities to establish coordination between public and private service providers and determine referral pathways. In addition, in these recommendations, the need for coordination within the system to ensure proper referral for testing, isolation and admission to the hospital is also mentioned (69).

In the next sub-theme, coordination between the MOHME and other organizations is mentioned. Other studies have pointed out that the scale of the pandemic requires coordination of efforts across government sectors as well as non-governmental organizations. For the health sector, this means horizontal (with other ministries, with relevant non-governmental actors and across borders) and vertical (at central, regional, and municipal levels) coordination in decision-making. Horizontal and vertical coordination seems necessary for aligning policymaking and implementation (70). Another study explored many shortcomings in the global response to the COVID-19 pandemic including the failure to coordinate efforts across geographic regions, within and between countries, ranks near the top of the list of poor performance (71).

The need to gain public trust is another mentioned issue, which can bring people's participation and their greater compliance with the laws. A study compared Indonesia's president's admission of delayed public risk communication for fear of the economic cost with Iran, where secrecy has certainly contributed to a rapid increase in the death rate. This led to people's severe distrust of the government's reporting system and its response capacity (68). In another study, it is explicitly stated that “your government needs you.” In this research, in response to the pandemic, the necessity of designing technology systems has been pointed out, and in the design of these systems, the necessity of the participation of people who use the systems daily has been pointed out (71).

### Recruiting, managing, and empowering health human resources with attention to financial and non-financial incentives

The effective provision of health services is strongly influenced by human resources. Results of this research showed that management of these valuable resources is one of the necessities that should be given more attention. Another study has also stated that one of the main challenges of healthcare systems against pandemic management was the challenge of recruitment, inappropriate number, type, distribution method and optimal management of human resources (11).

Inadequate knowledge of health workers is mentioned which comes from two sources. The first is that in Iran's healthcare system, education related to disaster health had been forgotten for many years. Second, specific training related to the current pandemic was also provided in a weak manner. Based on the published research at the beginning of the pandemic, a significant number of health workers had poor knowledge of the ways of transmission of the disease and the initial clinical symptoms (72, 73). In general, insufficient preparation, lack of specialized knowledge, and lack of access to practical skills were among the challenges of health workers in dealing with COVID-19 pandemic (11).

In the next two sub-themes, the financial and non-financial support of the health workforce is mentioned. One of the most important challenges in the field of non-financial issues is mental health problems. Evidence suggests that personnel working directly in the COVID-19 units have experienced many psychological changes due to unpredictable conditions, high workload, unknown nature of the disease, frequent changes in protocols and policies (72, 74). And in general, during the COVID-19 pandemic, negative psychological effects such as stress, depression, anxiety, insomnia, and feelings of anger have increased among healthcare workers (75). Using mental health consults and support for health workers during disasters, and empowering personnel's skills in managing stress and negative emotions can help reduce mental disorders (11). In the field of financial issues, delays in payments and lack of sufficient financial incentives were among the problems that need to be addressed. Similarly, another study has pointed out that public hospitals have faced many problems that led to a reduction of their financial ability to provide support facilities for personnel (76). It has been shown that in Colombia many hospitals have been forced to lay off their staff due to increased costs and loss of income during COVID-19 (77). On the other hand, nurses in Iran have also complained about the delay in payment of service compensation and the small payments they have received regarding their heavy duties (72).

### Reforming educational approaches in medical students' training

Another issue that needs to be addressed more is the training of students in practical work fields. Due to the unique structure of Iran's medical science system -a combined system of education, research, and service provision in universities of medical sciences- remarkable achievements can be made in the training of a skilled workforce that has experience working at the bedside since the student days (78). While it seems that in some fields of study, including health fields, this issue has not been realized as it should be.

Another issue that has been discussed for years, but practically nothing has been done about it, is the updating of the educational protocols of most academic fields. Several studies have been conducted on the impact of the pandemic on medical education (79, 80). Much evidence indicates that the usual methods should be changed, and training should be adjusted based on health protocols (81). Another study has pointed to effects and potentials of COVID-19 on medical education which led to revise the possibility of online learning, raise standards in medical education, and expand clinical learning (82).

### Lessons learned from neglected aspects

One of the most important unused potentials in Iran's healthcare system was the structure of the primary health care network. While in recommendations to strengthen the response to COVID-19, WHO has pointed out the importance of primary healthcare to deal more effectively with the pandemic and has introduced this network as a unique opportunity to increase the impact of many actions and an integral part of the public health response to COVID-19 (69).

Another weakness that needs more consideration was the adoption of inefficient decisions during the COVID-19 era, this weakness was especially observed in control policies. Others have argued that policies may be ineffective or even backfire unless they get everyone to act. The spread of COVID-19 has prompted governments and public health authorities to move toward restricting new infections. Most of these interventions rely on community compliance, and if not implemented strongly across all social groups, compliance will be impaired and the policy ineffective (83).

In the next sub-theme, it is mentioned that infrastructures and programs should be created to deal with the next possible pandemics. COVID-19 has highlighted the need for a more ambitious and sustainable approach to planning and a stronger infrastructure for preparedness. Similarly, others have stressed the need to plan for the next pandemic (71). Others also emphasize that although the consequences and casualties of COVID-19 are significant, lessons learned should be considered. Events like the current pandemic will occur again in the future, will have unpredictable characteristics, and will pose a great threat to all countries from a health, economic and social perspective. The only possible solution is to further strengthen the readiness of countries by obtaining political commitment and pre-planning (84).

## Conclusion

In summary, the present study revealed the need for a paradigm shift in various aspects of Iran's healthcare system. COVID-19 is not the first pandemic, and it will not be the last, and we will not be prepared for the next one unless we take bold steps. It is necessary to move toward e-health in step with advanced countries and provide the culture and infrastructure of evidence-based decision making. In addition, dedicated budgets for pandemics should be considered in the funding structure of MOHME, and by focusing on the challenges of providing services in the current pandemic, prevent this disruption in future outbreaks. Interactions at the national and international level should be given more attention and the power tools of MOHME, as the custodian of pandemic control, should be strengthened. Also, healthcare workers as valuable resources in pandemic management should be given special attention from all financial, non-financial and educational aspects. Furthermore, the effective training of future human resources, current students, should not be neglected either. And finally, one should think about which existing potentials have been neglected, apart from the basic weaknesses, and prepare for the next pandemics.

## Data availability statement

The original contributions presented in the study are included in the article/supplementary material, further inquiries can be directed to the corresponding author.

## Ethics statement

The article's proposal was approved by Ethics Committee affiliated with Shiraz University of Medical Sciences with the ID of IR.SUMS.REC.1399.1038. The informed consent obtained both in written form and verbally before starting interview and the Ethics Committee approved this procedure with the above ethical code consent for publication. The patients/participants provided their written informed consent to participate in this study.

## Author contributions

MM: study design, data collection, data analysis, accrual of study participants, and writing and reviewing the manuscript. PB: study design, data analysis, accrual of study participants, reviewing the manuscript for critical revisions, and important intellectual content. SD: data analysis, accrual of study participants, and critically revising the manuscript for important intellectual content. ZK and MP: data analysis and accrual of study participants. RI: translation of the article and correction of grammar and language, data analysis, and reviewing the manuscript for critical revisions. All authors read and approved the final version of the manuscript.
